# Molecular characteristics of extended-spectrum β-lactamase-producing *Escherichia coli* isolated from the rivers and lakes in Northwest China

**DOI:** 10.1186/s12866-018-1270-0

**Published:** 2018-10-04

**Authors:** Haixia Liu, Hongchao Zhou, Qinfan Li, Qian Peng, Qian Zhao, Jin Wang, Xiaoqiang Liu

**Affiliations:** 1 Department of Aquaculture, College of Animal Science and Technology, Northwest A&F University, Yangling, 712100 Shaanxi China; 2 Department of Basic Veterinary, College of Veterinary Medicine, Northwest A&F University, Yangling, 712100 Shaanxi China

**Keywords:** *Escherichia coli*, Surface water, Antibiotic resistance, β-Lactamase, PMQR

## Abstract

**Background:**

Extended-spectrum β-lactamases (ESBLs)-producing *Escherichia coli* (*E. coli*) isolates in environment water become progressively a potential threat to public health, while the detailed information about the ESBL-producing *E. coli* isolates in the rivers and lakes in Northwest China is scarce. In the present study, it was aimed to characterize the ESBL-producing *E. coli* isolated from the surface waters in Northwest China.

**Results:**

A total of 2686 *E. coli* isolates were obtained from eleven rivers and lakes in Northwest China to screen for ESBL producers. Seventy-six (2.8%) isolates were classified as ESBL producers, and phylogenic groups D and A accounted for 59.2% of the ESBL producers. CTX-Ms were the predominant ESBLs genotype, and they were represented by seven *bla*_CTX-M_ subtypes. *bla*_CTX-M-14_ was the most prevalent specific CTX-M gene, followed by *bla*_CTX-M-9_, *bla*_CTX-M-123_, *bla*_CTX-M-15_, *bla*_CTX-M-27,_ *bla*_CTX-M-1_ and *bla*_CTX-M-65_. Moreover, 54 of the 76 ESBL producers carried at least one plasmid-mediated quinolone resistance (PMQR) gene, and *aac(6′)-Ib-cr* was predominant. The overall occurrence of virulence factors ranged from 1.3% (*eae*) to 48.7% (*traT*). Thirty-seven sequence types (STs) were confirmed among the 76 ESBL producers, and the predominant was ST10, which was represented by 10 isolates; importantly, clone B2-ST131, associated with severe infections in humans and animals, was detected three times.

**Conclusion:**

The prevalence of ESBL-producing *E. coli* from the rivers and lakes in Northwest China was low (2.8%), and the extraintestinal pathogenic *E. coli* (ExPEC) pathotype was the most commonly detected on the basis of the virulence factor profiles. 76.3% of ESBL producers harbored more than one β-lactamase gene, and *bla*_CTX-M-14_ was the predominant genotype. Notably, one ST131 isolate from Gaogan Canal simultaneously harbored *bla*_CTX-M-9_, *bla*_CTX-M-15_, *bla*_CTX-M-123_, *bla*_KPC-2_, *bla*_NDM-1_, *bla*_OXA-2_ as well as the PMQR genes *qnrA*, *qnrS* and *aac(6′)-Ib-cr*.

## Background

The use of a wide variety of antimicrobials in human medicine, veterinary clinics, livestock industries and aquaculture has resulted in the emergence and spread of antibiotic-resistant bacteria in different environments, particularly in many developing countries [1, 2]. It becomes evident that the resistance genes can be introduced into the natural bacterial community as the antibiotic-resistant bacteria in humans and animals entered the water bodies [3]. Hence, it is necessary to clarify the potential threat associated with the occurrence of antibiotic-resistant bacteria in water environments in order to further evaluate public health risk and prevent waterborne infections. As one of the most typical indicator bacterium of fecal contamination in the environments, *Escherichia coli* (*E. coli*) can easily acquire resistance to antibiotics consumption in humans and animals [4]. Generally, pathogenic *E. coli* isolates were categorized into several pathotypes based on the clinical symptoms of the patients and the distinct virulence traits of the bacteria. Therefore, *E. coli* isolates are characterized by their virulence properties and mechanisms of pathogenicity into the enteropathogenic *E. coli* (EPEC), enterotoxigenic *E. coli* (ETEC), shiga toxin-producing *E. coli* (STEC), enteroinvasive *E. coli* (EIEC), enteroaggregative *E. coli* (EAEC) as well as extraintestinal pathogenic *E. coli* (ExPEC) [5, 6]. STEC isolates are defined as *E. coli* isolates expressing either *stx*_*1*_ or *stx*_*2*_; EPEC isolates are defined as *eae*-harboring diarrheagenic *E. coli* isolates that do not possess the *stx* gene; ETEC isolates are characterized by *estA* and *eltB*; isolates carrying *aggR* and *ipaH* are referred to as EAEC and EIEC, respectively [7]. Lastly, ExPEC isolates are associated with *fyuA*, *iutA*, *afa*, *papA*, *focG*, *sfaS*, *kpsM*II, *hlyD* and *traT*. Thus, the pathotypes of the uncharacterized isolates can be inferred from their virulence properties.

Since the extended-spectrum β-lactamases (ESBLs) was firstly reported in 1979 [8], the prevalence of ESBL-producing bacteria have been frequently detected worldwide from clinical isolates due to the increasing use of β-lactam antibiotics and carbapenems; the latter are usually used as the last resort for most serious bacterial infections. Moreover, some ESBL-producing isolates have been recovered from surface waters, where contamination from unmetabolized antibiotics may exert a selective pressure on bacteria, resulting in the emergence and spread of antibiotic-resistant isolates, especially the multidrug-resistant (MDR) isolates during their migration in water resources [3]. Relatedly, plasmid-mediated quinolone resistance (PMQR) determinants also pose a serious threat to public health, and some PMQR genes are considered to be associated with the ESBLs encoding genes [9]. The spread of *E. coli* co-expressing quinolone resistance along with ESBLs into rivers and lakes is worrisome and contributes to the growing concerns about resistant *E. coli* and their potential hazards to the environment.

Until now, little data are available on the ESBL-producing *E. coli* isolates in the surface waters in Northwest China. Thus, the current study was designed to gain insight into the prevalence of ESBL-producing *E. coli* isolates obtained throughout March 2015 to November 2016 from the rivers and lakes in Shaanxi province, and to further analyze the molecular characteristics of the ESBL producers.

## Methods

### Collection of isolates

**Fig. 1:**
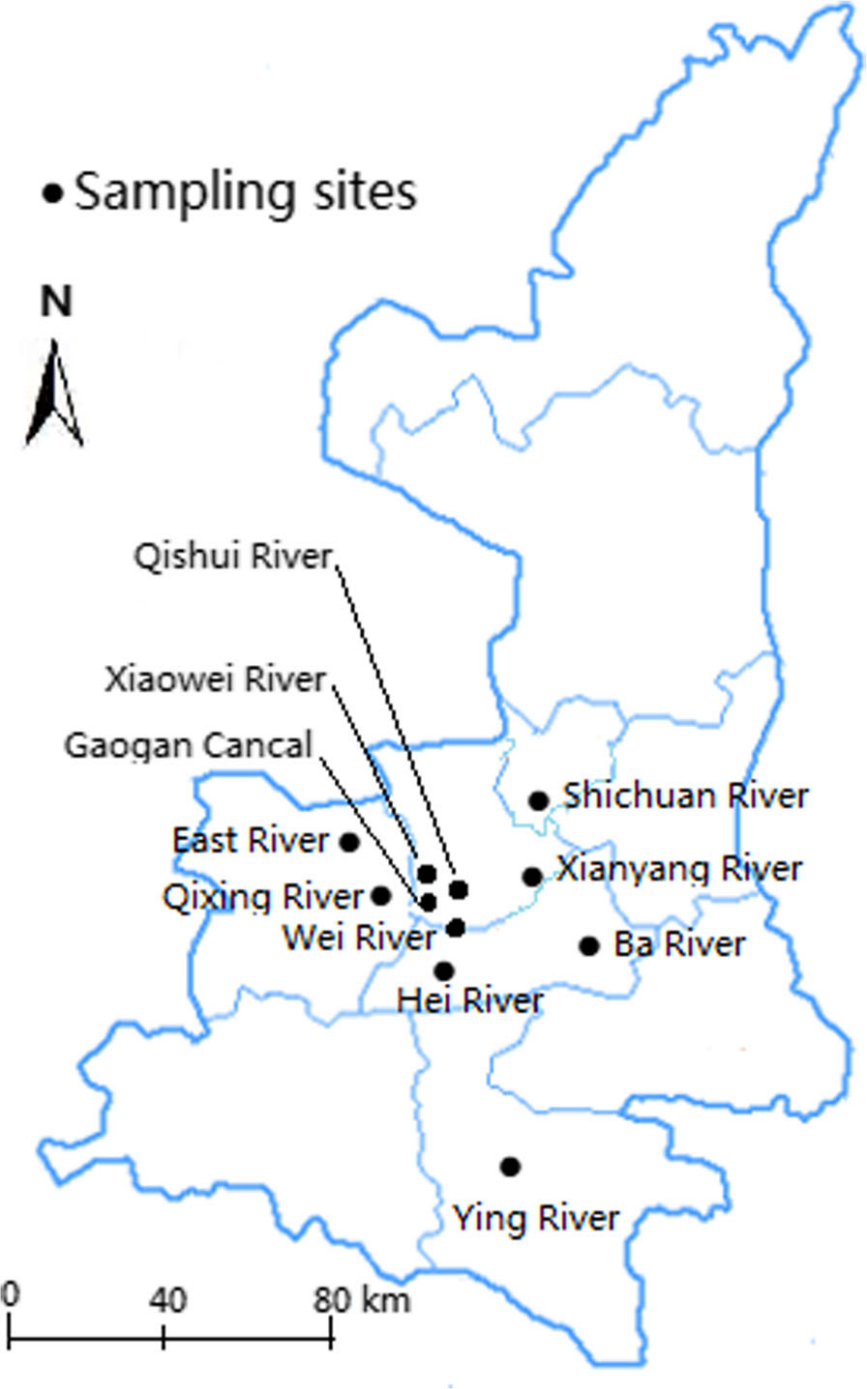
The map of sample locations

Between March 2015 and November 2016, a total of 2686 *E. coli* isolates were obtained from eleven water bodies located in Shaanxi province, Northwest China, including Hei River (*n* = 177), Ying Lake (*n* = 194), Xianyang Lake (*n* = 196), Qishui River (*n* = 264), East Lake of Fengxiang county (*n* = 154), Wei River (*n* = 343), Ba River (*n* = 256), Shichuan River (*n* = 294), Xiaowei River (*n* = 265), Qixing River (*n* = 276) and Gaogan Canal of Yangling (*n* = 267) (Fig. 1). Among these water bodies, Hei River functioned as a public water supply source, while the others were scenic spots or functioned as floodways of the cities and countryside. All sampling sites were sampled once or multiple times, and all samples were collected in sterile 500-ml polyethylene bottles without preservatives and transported at 4 °C to the Veterinary Pharmacology Laboratory in Northwest A&F University, where primary isolation of *E. coli* was performed. Briefly, multiple volumes of untreated water were membrane filtered directly through 0.45-μm pore size filters, and the filters were placed on MacConkey agar plates (Solarbio Science & Technology, Co., Ltd., Beijing, China) at 37 °C for the identification of *E. coli* isolates. All 2686 putative *E. coli* colonies on MacConkey agar were restreaked onto Eosin Methylene Blue agar (Solarbio Science & Technology, Co., Ltd., Beijing, China), and then the suspicious colonies of *E. coli* were further identified with standard biochemical tests. Finally, the confirmed isolates as *E. coli* were stored at − 80 °C in Tryptic Soy broth (Solarbio Science & Technology, Co., Ltd., Beijing, China) containing 30% glycerol until use.

### Antimicrobial susceptibility testing

The broth microdilution procedure recommended by Clinical Laboratories Standards Institute (CLSI) [10] was performed to determine the antimicrobial susceptibility of all *E. coli* isolates against 16 antimicrobials representing six antimicrobial classes: β-lactams, including penicillins (ampicillin, amoxicillin-clavulanic acid and ticarcillin-clavulanic acid), the first-generation cephalosporins (cephalothin), the third-generation cephalosporins (cefotaxime, ceftazidime and ceftriaxone), cephamycins (cefoxitin), and carbapenems (meropenem); tetracyclines (tetracycline); amphenicols (thiamphenicol); quinolones (nalidixic acid and ciprofloxacin); aminoglycosides (gentamicin and amikacin); sulfonamides (sulfamethoxazole-trimethoprim). The control strain for susceptibility testing was *E. coli* ATCC 25922.

Moreover, ESBL production among the *E. coli* isolates resistant to the third-generation cephalosporins was detected phenotypically by the double disk synergy test with disks supplemented with cefotaxime and ceftazidime alone or coupled with clavulanic acid [10]. Initial screening analyses indicated that 2.8% (*n* = 76) *E. coli* isolates were phenotypic ESBL-positive isolates, and these isolates were used for further analysis.

### Phylogenetic typing and determination of virulence factors

Total DNA was isolated from the ESBL producers by using the boiling method. Phylogenetic grouping was determined for the ESBL-producing isolates according to the novel quadruplex PCR method [11]. Meanwhile, seven virulence factor genes known to be characteristic of intestinal pathogenic *E. coli* (IPEC), including *aggR* for EAEC, *stx*_*1*_ and *stx*_*2*_ for STEC; *eae* for EPEC, *estA* and *eltB* for ETEC, EIEC-specific gene *ipaH*; as well as seven markers of virulence associated with uropathogenic *E. coli* (UPEC), including *traT*, *fyuA*, *papC*, *chuA*, *afa/dra*, *iutA* and PAI [12], were performed by PCR.

### Characterization of β-lactamase and PMQR genes

PCR detection and gene identification were performed for β-lactamase genes (TEM, SHV, CTX-Ms), plasmid-mediated AmpC β-lactamase (CMY-2) and carbapenemase genes (class A, KPC-2; class B, NDM-1; class D, OXA) in ESBL-producing *E. coli*. *bla*_CTX-M_ group-specific primers for CTX-M-1, CTX-M-2, CTX-M-8 and CTX-M-9 were used to detect of *bla*_CTX-M_ genes. The PCR products were purified and sequenced by Sangon Biotech (Shanghai, China), and then the β-lactamase genes were identified using the β-lactamase database (http://www.lahey.org/studies/webt.asp) after all the sequences were analyzed online using BLAST (http://blast.ncbi.nlm.nih.gov/Blast.cgi). Moreover, all the 76 ESBL-producing *E. coli* isolates were screened by PCR for PMQR genes (*qnrA*, *qnrB*, *qnrD*, *qnrS*, *aac(6′)-Ib-cr*, *oqxAB* and *qepA*) as described previously [13, 14].

### Conjugation experiments

Potential horizontal transferability of β-lactamase and PMQR genes from 15 randomly selected ESBL-producing *E. coli* isolates (at least one isolate per sampling site) was assessed by conjugation studies (broth mating method) using *E. coli* J53 AZ^r^ as the recipient [15]. The Mueller-Hinton agar supplemented with 150 μg/ml sodium azide and 2 μg/ml cefotaxime were used to select the transconjugants, which were subsequently analyzed by PCR to determine the transferability of β-lactamase and PMQR genes. In addition, the resistance patterns of the recipient and all transconjugants were analyzed.

### Multilocus sequence typing (MLST) determination

Internal fragments of seven conserved housekeeping genes (*adk*, *fumC*, *gyrB*, *icd*, *mdh*, *purA* and *recA*) of each ESBL-producing *E. coli* isolate were amplified by PCR. A detailed scheme of the MLST procedure, including the primers, PCR conditions, allelic type and sequence type assignment methods, is available at MLST database website (http://mlst.warwick.ac.uk/mlst/dbs/Ecoli).

### Statistical analysis

Pearson′s Chi-squared test was used for statistical analysis, and the statistical significance level was established at *P* < 0.05.

## Results

### Antimicrobial susceptibility

Among the 2686 *E. coli* isolates collected, 76 (2.8%) isolates were identified as the ESBL-producing isolates, which were unevenly distributed in 11 sampling sites at levels ranging from 1.1 to 6.4%. Moreover, 64 of the 76 (84.2%) isolates expressed the MDR phenotype. The 76 ESBL-producing isolates showed high resistance to tetracycline (97.3%), followed by ticarcillin-clavulanic acid (90.8%), cephalothin (89.5%), nalidixic acid (81.6%), cefotaxime (77.6%), ciprofloxacin (69.7%), sulfamethoxazole-trimethoprim (69.7%), thiamphenicol (63.2%), and cefoxitin (57.6%), whereas they exhibited high susceptibility to meropenem (96.1%).

### Phylogenetic groups and the virulence genes distribution

Phylogenetic analysis showed that the 76 ESBL-producing isolates were composed of phylogenetic groups D (*n* = 24), A (*n* = 21), B2 (*n* = 15), B1 (*n* = 10), C (*n* = 4), and E (*n* = 2). Overall, 78.9% (60/76) of ESBL-producing isolates harbored as least one virulence factor, and the prevalence of individual virulence genes ranged from 1.3% (*eae*) to 52.6% (*traT*). *estA* and *aggR* were detected in ten and two isolates, respectively, while *stx1*, *stx2* and *ipaH* were not detected. The virulence genes associated with UPEC isolates were detected throughout the sources, whereas the virulence genes associated with STEC and EIEC isolates were not detected.

### Distribution of β-lactamase and PMQR genes

As shown in Table 1, *bla*_SHV_, *bla*_TEM_ and *bla*_CTX-M_ were detected in 36.8% (*n* = 28), 43.4% (*n* = 33) and 76.3% (*n* = 58) of ESBL producers, respectively, and 58 of the 76 isolates possessed more than one β-lactamase gene. It is interesting that the number of the β-lactamase genes in an *E. coli* isolate was positively correlated the prevalence of the ESBL producer in each sampling site. For the *bla*_CTX-M_ positive isolates, *bla*_CTX-M-14_ (*n* = 35) was the predominant genotype, followed by *bla*_CTX-M-9_ (*n* = 17), *bla*_CTX-M-123_ (*n* = 15), *bla*_CTX-M-15_ (*n* = 7), *bla*_CTX-M-27_ (*n* = 4), *bla*_CTX-M-1_ (*n* = 3) and *bla*_CTX-M-65_ (*n* = 3). On the other hand, *bla*_OXA-2_, *bla*_KPC-2_, *bla*_CMY-2_ and *bla*_NDM-1_ were detected in five, four, two and one isolate, respectively. It is noteworthy that 80% (4/5) of *bla*_OXA-2_ positive isolates were isolated from Gaogan Canal. Among the 33 TEM-positive isolates, two were *bla*_TEM-3_ and the rest were non-ESBL gene *bla*_TEM-1_. The *bla*_SHV_ genes were represented by *bla*_SHV-2_ (*n* = 7) and *bla*_SHV-12_ (*n* = 21), and it is interesting to note that ESBL gene *bla*_SHV-12_ and non-ESBL gene *bla*_TEM-1_ simultaneously appeared in 20 isolates. Furthermore, 54 of 76 (71.1%) ESBL-producing isolates harbored at least one PMQR gene, which was co-located in the ESBL producers with β-lactamase genes. *Aac(6′)-Ib-cr* (*n* = 46) was the most dominant PMQR gene, followed by the *qnr* genes (*n* = 34). Moreover, one isolate harbored the *qepA* gene, while the *oqxAB* gene was not detected in any isolate.

**Table 1:** ESBL-producing *E. coli* isolates from rivers and lakes in the Northwest China

Sampling sites	Isolates No.	PG	Antimicrobial resistance profiles	β-lactamase genes	PMQR genes	Virulence genes	MLST
Hei River	HH1609014	B1	AMP AMC TIM CEP CTX CEX FOX TEC GEM AMK SXT	CTX-M-14		*fyuA*, *traT*	ST155
	HH1510025	B2	AMP CEP TPH GEM SXT	TEM-1, SHV-12	*qnrB*		ST1587
Ying Lake	YH1507022	A	AMP AMC TIM CEP CTX CAZ CEX FOX TEC TPH NAC CIP SXT	TEM-1, CTX-M-14	*aac(6′)-Ib-cr*	*traT*, *papC*, *chuA*	ST617
	YH1606032	A	AMP AMC TIM CEP CTX CAZ CEX FOX TEC NAC CIP GEM AMK SXT	CTX-M-14	*aac(6′)-Ib-cr*	*afa/dra*, PAI	ST44
	YH1607018	D	AMP AMC TIM CEP CAZ TEC TPH GEM AMK	TEM-1, SHV-12		*traT*, *chuA*	ST2148
Xianyang Lake	XY1608045	B2	AMP AMC TIM CEP CTX CAZ TEC TPH NAC CIP GEM AMK SXT	CTX-M-14	*aac(6′)-Ib-cr*	*traT*, *iutA*	ST602
	XY1605044	D	AMP AMC TIM CEP CAZ CEX FOX TEC	CTX-M-14			ST393
	XY1605033	D	AMP AMC TIM CEP CEX TEC NAC CIP SXT	TEM-1, SHV-12	*qnrB*, *aac(6′)-Ib-cr*	*traT*, *chuA*	ST393
	XY1507042	E	AMP AMC CTX CAZ TPH NAC CIP	TEM-1, SHV-12		*fyuA*, *traT*	ST1301
Qishui River	QS1608021	A	AMP AMC TIM CEP CTX CEX FOX TEC NAC CIP SXT	TEM-1, CTX-M-1	*aac(6′)-Ib-cr*	*estA*	ST10
	QS1607026	A	AMP AMC TIM CEP CTX CAZ TEC NAC CIP GEM SXT	CTX-M-9		*traT*, *chuA*	ST4429
	QS1608034	B2	AMP AMC TIM CEP CEX FOX TEC TPH NAC CIP SXT	CTX-M-9	*qnrB*, *qnrS*	*traT*, *chuA*	ST331
	QS1610030	C	AMP AMC TIM CEP CEX TEC TPH NAC CIP	TEM-1, SHV-12	*qnrB*, *aac(6′)-Ib-cr*	*estA*	ST23
East Lake	EH1507029	A	AMP AMC CTX CAZ TEC NAC CIP SXT	CTX-M-1	*qnrB*, *aac(6′)-Ib-cr*		ST10
	EH1607033	A	AMP AMC TIM CTX CAZ CEX FOX TEC SXT	CTX-M-14	*aac(6′)-Ib-cr*	*traT*, *chuA*, *papC*,	ST10
	EH1607014	A	AMP AMC TIM CEP CTX CAZ CEX FOX TEC TPH NAC CIP GEM SXT	TEM-1, CTX-M-14	*aac(6′)-Ib-cr*	*traT*, PAI	ST167
	EH1608016	A	AMP AMC TIM CEP CAZ TEC NAC CIP	SHV-12	*aac(6′)-Ib-cr*		ST167
Wei River	WH1606023	A	AMP AMC CEP CEX FOX TEC TPH	TEM-1, SHV-12	*aac(6′)-Ib-cr*	*fyuA*	ST10
	WH1508055	B1	AMP AMC TIM CTX CAZ TEC NAC CIP GEM AMK SXT	CTX-M-27	*aac(6′)-Ib-cr*	*traT*, *afa/dra*	ST58
	WH1606078	D	AMP AMC TIM CEP CEX FOX TEC TPH NAC CIP GEM AMK SXT	CTX-M-14	*qnrS*	*traT*, *papC*, *afa/dra*	ST609
	WH1510002	D	AMP AMC TIM CEP CTX CAZ CEX	CTX-M-9	*qnrB*, *aac(6′)-Ib-cr*	*fyuA*, *traT*, *papC*	ST38
	WH1607120	E	AMP AMC TIM CEP CTX CAZ TEC NAC CIP GEM AMK SXT	CTX-M-14	*aac(6′)-Ib-cr*	*traT*	ST1301
Ba River	BA1605012	A	AMP AMC TIM CEP TEC TPH NAC CIP SXT	TEM-1, SHV-12	*aac(6′)-Ib-cr*		ST44
	BA1605022	B1	AMP AMC TIM CEP CTX CEX TEC NAC CIP SXT	CTX-M-9, CTX-M-14	*qnrS*, *aac(6′)-Ib-cr*	*traT*, *afa/dra*	ST155
	BA1508024	D	AMP AMC TIM CEP CAZ TEC NAC	TEM-1, SHV-12			ST4068
	BA1510031	D	AMP AMC TIM CEP CTX CAZ CEX FOX TEC TPH SXT	CTX-M-9, CTX-M-14		*traT*, *papC*, *sfaS*	ST2003
	BA1509025	D	AMP AMC TIM CTX CAZ CEX TEC TPH NAC CIP GEM AMK	CTX-M-14, CTX-M-15	*qnrB*, *aac(6′)-Ib-cr*	*fyuA*, *traT*, *papC*	ST69
	BA1509015	D	AMP AMC TIM CTX CEX FOX TEC TPH	CTX-M-14, CTX-M-15	*qnrS*, *aac(6′)-Ib-cr*	*fyuA*, *iutA*	ST405
Shichuan River	SC1608022	A	AMP AMC TIM CEP CTX CAZ TEC SXT	SHV-12, CTX-M-123		*traT*, *chuA*	ST93
	SC1506012	A	AMP AMC TIM CEP CTX CAZ CEX FOX TEC TPH NAC SXT	CTX-M-14			ST746
	SC1507014	A	AMP AMC TIM CEP CTX CAZ CEX FOX TEC NAC CIP SXT	TEM-3, CTX-M-123	*aac(6′)-Ib-cr*	*traT*, *papC*, *hlyD*	ST2376
	SC1604029	B1	AMP AMC TIM CEX FOX TEC TPH NAC CIP GEM AMK	TEM-1, SHV-12	*qnrS*, *aac(6′)-Ib-cr*		ST155
	SC1607063	B2	AMP AMC TIM CEP CTX CEX FOX MEM TEC TPH NAC CIP GEM AMK SXT	CTX-M-15, CTX-M-123	*qnrS*, *aac(6′)-Ib-cr*	*fyuA*, *traT*, *papC*	ST131
	SC1608102	B2	AMP AMC TIM CEP CTX CAZ CEX TIC TPH NAC	TEM-1, SHV-2		*fyuA*	ST95
	SC1610005	D	AMP AMC TIM CEP CTX CEX FOX TEC NAC CIP GEM AMK SXT	TEM-1, SHV-12, CTX-M-15	*qnrS*, *aac(6′)-Ib-cr*	*estA*	ST38
	SC1609081	D	AMP AMC TIM CEP CTX CEX TEC NAC CIP GEM SXT	TEM-1, CTX-M-14	*qnrB*, *aac(6′)-Ib-cr*	*estA*	ST405
Xiaowei River	XW1608112	A	AMP AMC TIM CEP CTX CEX FOX TEC TPH NAC CIP GEM AMK SXT	TEM-1, SHV-12	*qnrB*, *aac(6′)-Ib-cr*	*iutA*, *afa/dra*	ST10
	XW1608047	A	AMP AMC TIM CEP CTX CAZ CEX FOX TEC NAC SXT	TEM-1, SHV-12, CTX-M-14			ST44
	XW1609034	B1	AMP AMC TIM CEP CTX CAZ CEX TEC TPH NAC CIP SXT	CTX-M-14, CTX-M-65	*qnrS*	*fyuA*, PAI	ST75
	XW1608023	B2	AMP AMC TIM CEP CTX CEX TEC TPH NAC	TEM-1, SHV-2		*traT*, *chuA*	ST95
	XW1607012	B2	AMP AMC TIM CEP CTX CAZ CEX FOX TEC TPH NAC CIP SXT	CTX-M-9, CTX-M-14, CTX-M-123	*aac(6′)-Ib-cr*	*fyuA*, *traT*, *iutA*	ST12
	XW1607055	B2	AMP AMC TIM CEP CTX CAZ FOX TEC TPH NAC CIP SXT	CTX-M-14			ST2855
	XW1609057	D	AMP AMC TIM CEP CTX CAZ TEC TPH SXT	TEM-1, SHV-12			ST5164
	XW1608026	D	AMP AMC TIM CEP CTX CAZ CEX FOX TEC NAC CIP GEM AMK SXT	CTX-M-1	*aac(6′)-Ib-cr*	*traT*, *hlyD*	ST3880
	XW1607034	D	AMP AMC TIM CEP CTX CAZ CEX FOX TEC NAC CIP SXT	CTX-M-14, CTX-M-123	*aac(6′)-Ib-cr*	*fyuA*, *traT*	ST38
	XW1609038	D	AMP AMC TIM CEP CTX CEX FOX MEM TEC NAC CIP GEM AMK SXT	CTX-M-15	*qnrB*, *aac(6′)-Ib-cr*	*traT*, *iutA*, *papC*	ST69
	XW1608041	D	AMP AMC CEP CTX CEX TEC TPH NAC CIP SXT	TEM-1, SHV-2, CTX-M-14		*traT*, *chuA*	ST609
Qixing River	QX1608021	A	AMP AMC TIM CEP CTX CAZ TEC TPH NAC SXT	TEM-1, SHV-12			ST10
	QX1608013	A	AMP AMC TIM CEP CEX TEC TPH SXT	TEM-1, SHV-12	*aac(6′)-Ib-cr*	*traT*, *papC*, PAI	ST10
	QX1509072	A	AMP AMC TIM CEP CTX CAZ FOX TEC NAC GEM AMK SXT	TEM-1, SHV-2	*qnrS*, *aac(6′)-Ib-cr*	*estA*	ST10
	QX1608015	A	AMP AMC TIM CEP CTX CEX FOX TEC TPH NAC CIP GEM AMK SXT	CTX-M-9, CTX-M-27	*qnrS*	*traT*, *papC*	ST3902
	QX1605083	B1	AMP AMC TIM CEP CTX CAZ CEX FOX TEC TPH NAC CIP GEM AMK SXT	TEM-1, CTX-M-9, KPC-2		*fyuA*, *papC*, *traT*	ST3160
	QX1608005	B1	AMP AMC TIM CEP CTX CAZ CEX TEC NAC CIP SXT GEM SXT	CTX-M-14	*qnrB*	*estA*	ST75
	QX1507055	B2	AMP AMC TIM CEP CTX CAZ CEX FOX TEC TPH NAC CIP GEM SXT	CTX-M-27	*qnrS*	*aggR*	ST1304
	QX1508112	B2	AMP AMC TIM CEP CTX CAZ FOX TEC TPH NAC CIP GEM AMK SXT	TEM-1, SHV-12, CTX-M-9, OXA-2	*qnrB*, *qnrS*, *aac(6′)-Ib-cr*	*traT*, *iutA*, PAI	ST12
	QX1608059	B2	AMP AMC CEP CAZ CEX FOX TEC TPH NAC CIP GEM AMK	CTX-M-14, CTX-M-123		*estA*	ST2077
	QX1510043	C	AMP AMC TIM CEP CTX CAZ CEX FOX TEC NAC CIP GEM SXT	CTX-M-9, CTX-M-14	*aac(6′)-Ib-cr*	*traT*, *papC*	ST23
	QX1608046	D	AMP AMC TIM CEP CTX CAZ CEX FOX TEC TPH NAC CIP SXT	CTX-M-14, CTX-M-123, CTX-M-65		*afa/dra*, *hlyD*	ST3880
	QX1604103	D	AMP AMC TIM CEP CEX FOX TEC TPH NAC CIP GEM AMK SXT	TEM-1, SHV-2, CTX-M-14, CTX-M-123	*qnrB*, *aac(6′)-Ib-cr*	*fyuA*, *iutA*, PAI	ST609
	QX1609108	D	AMP AMC TIM CEP CTX CAZ CEX FOX MEM TEC TPH NAC CIP SXT	TEM-3, CTX-M-14		*fyuA*, *afa/dra*	ST2148
Gaogan Canal	GG1505017	A	AMP AMC TIM CEP CTX CEX TEC NAC CIP GEM AMK SXT	TEM-1, SHV-2, CTX-M-14	*aac(6′)-Ib-cr*		ST10
	GG1509025	A	AMP AMC TIM CEP CEX TEC TPH GEM AMK SXT	TEM-1, SHV-12, CTX-M-65	*aac(6′)-Ib-cr*	*estA*	ST10
	GG1508074	B1	AMP AMC TIM CEP CTX CAZ CEX MEM TEC TPH NAC CIP SXT	TEM-1, SHV-12, CTX-M-9	*aac(6′)-Ib-cr*	*eae*	ST58
	GG1609024	B1	AMP AMC TIM CEP CTX CAZ CEX MEM TEC TPH NAC CIP SXT	CTX-M-9, CTX-M-123	*qnrB*	*traT*, *papC*, *afa/dra*	ST155
	GG1609158	B1	AMP AMC CEP CEX FOX TEC TPH N GEM SXT	TEM-1, SHV-12			ST1049
	GG1609019	B2	AMP AMC TIM CEP CTX CAZ FOX TEC TPH SXT	CTX-M-9, CTX-M-14		*iutA*, *afa/dra*	ST3252
	GG1609022	B2	AMP AMC TIM CEP CTX CAZ CEX FOX TEC NAC CIP	CTX-M-9, CTX-M-14	*qnrA*, *aac(6′)-Ib-cr*	*fyuA*, *traT*, *iutA*, PAI	ST12
	GG1609068	B2	AMP AMC CEP CTX CEX FOX TEC NAC CIP SXT	CTX-M-14, KPC-2, OXA-2	*qnrB*, *qnrS*, *aac(6′)-Ib-cr*	*fyuA*, *papC*, *traT*, *iutA*	ST131
	GG1610109	B2	AMP AMC TIM CEP CTX CAZ CEX FOX MEM TEC TPH NAC CIP GEM AMK SXT	CTX-M-9, CTX-M-15, CTX-M-123, KPC-2, NDM-1, OXA-2	*qepA*, *qnrS*, *aac(6′)-Ib-cr*	*fyuA*, *papC*, *traT*, *chuA*, *iutA*	ST131
	GG1609086	C	AMP AMC TIM CEP CTX CAZ FOX TEC TPH NAC CIP SXT	CTX-M-9, CTX-M-14, CTX-M-123	*qnrS*, *aac(6′)-Ib-cr*	*traT*, *afa/dra*, *papC*, PAI	ST410
	GG1607066	C	AMP AMC TIM CEP CTX CAZ CEX TEC TPH NAC CIP GEM SXT	TEM-1, SHV-12, CTX-M-123	*qnrB*, *aac(6′)-Ib-cr*	*traT*, *chuA*	ST88
	GG1609121	D	AMP AMC TIM CEP CTX CAZ TEC TPH NAC CIP GEM AMK SXT	CTX-M-15, CTX-M-123	*aac(6′)-Ib-cr*	*estA*	ST38
	GG1609016	D	AMP AMC TIM CEP CTX CAZ CEX FOX TEC TPH NAC SXT	CTX-M-14, CMY-2	*aac(6′)-Ib-cr*	*fyuA*, *traT*	ST69
	GG1604028	D	AMP AMC TIM CTX CEX FOX TEC NAC CIP GEM AMK	CTX-M-14, CTX-M-123	*aac(6′)-Ib-cr*	*aggR*	ST69
	GG1506027	D	AMP AMC TIM CEP CTX CAZ CEX FOX TEC TPH NAC CIP GEM AMK SXT	CTX-M-9, CTX-M-123, KPC-2, OXA-2	*qnrB*, *aac(6′)-Ib-cr*	*fyuA*, *traT*, *chuA*, *iutA*	ST405
	GG1608063	D	AMP AMC TIM CEP CTX CAZ CEX FOX MEM TEC TPH NAC CIP SXT	CTX-M-14, CTX-M-27, CMY-2, OXA-2	*qnrS*, *aac(6′)-Ib-cr*	*fyuA*, *traT*, *chuA*, *iutA*, PAI	ST405

*AMP* ampicillin, *AMC* amoxicillin-clavulanic acid, *TIM* ticarcillin-clavulanic acid, *CEP* cephalothin, *CTX* cefotaxime, *CAZ* ceftazidime, *CEX* ceftriaxone, *FOX* cefoxitin, *MEM* meropenem, *TEC* tetracycline, *TPH* thiamphenicol, *NAC* nalidixic acid, *CIP* ciprofloxacin, *GEN* gentamicin, *AMK* amikacin, *SXT* sulfamethoxazole-trimethoprim

### Conjugation experiments

Ten out of fifteen ESBL producers were horizontally transferred to recipient strain *E. coli* J53 AZ^r^. PCR demonstrated the presence of β-lactamase and PMQR genes in transconjugants (Table 2). Antimicrobial susceptibility patterns revealed that all transconjugants kept the similar antibiotic resistance profiles to ampicillin, amoxicillin-clavulanic acid, ticarcillin-clavulanic acid, cefotaxime, ceftazidime, ceftriaxone and cefoxitin compared with the donors, and all transconjugants exhibited at least 8-fold increase in MICs compared with the recipient. The ciprofloxacin MICs for eight transconjugants harboring PMQRs ranged from 0.125 to 1 μg/ml, representing an increase of 2-fold to 16-fold compared with the recipient (Table 2). However, the transconjugants were still susceptible to meropenem, tetracycline, ciprofloxacin, gentamicin, thiamphenicol and sulfamethoxazole-trimethoprim.

**Table 2:** Antimicrobial susceptibility profiles of ESBL-producing *E. coli* isolates used in the conjugation experiments

Isolates	MIC (μg/ml) of antimicrobials													Presence of	
	AMP	AMC	TIM	CTX	CAZ	CEX	FOX	MEM	TEC	TPH	CIP	GEN	SXT	β-lactamase genes	PMQR genes
**Donors**															
HH1609014	256	32	32	32	4	32	8	0.03	32	2	0.5	32	128	CTX-M-14	
XY1608045	512	32	16	32	64	4	2	0.125	64	32	64	32	32	CTX-M-14	*aac(6′)-Ib-cr*
EH1607014	256	32	32	64	64	128	16	0.063	32	64	128	16	64	TEM-1, CTX-M-14	*aac(6′)-Ib-cr*
WH1510002	256	32	32	32	128	64	1	0.063	0.25	1	2	4	16	CTX-M-9	*qnrB*, *aac(6′)-Ib-cr*
BA1605022	512	64	16	64	8	64	2	0.063	64	0.25	32	2	64	CTX-M-9, CTX-M-14	*qnrS*, *aac(6′)-Ib-cr*
QX1604103	256	64	32	4	256	128	32	0.03	128	128	128	64	256	TEM-1, SHV-2, CTX-M-14, CTX-M-123	*qnrB*, *aac(6′)-Ib-cr*
SC1610005	512	64	32	32	8	64	32	0.03	32	2	16	32	128	TEM-1, SHV-12, CTX-M-15	*qnrS*, *aac(6′)-Ib-cr*
XW1609038	256	32	32	32	4	64	16	4	0.25	0.5	64	128	64	CTX-M-15	*qnrB*, *aac(6′)-Ib-cr*
GG1509025	256	64	16	4	2	32	2	0.125	128	64	2	64	64	TEM-1, SHV-12, CTX-M-65	*aac(6′)-Ib-cr*
GG1610109	512	64	32	128	64	128	32	16	128	128	128	128	128	CTX-M-9, CTX-M-15, CTX-M-123, KPC-2, NDM-1, OXA-2	*qepA*, *qnrS*, *aac(6′)-Ib-cr*
Recipient J53AZ^r^	4	1	1	0.125	0.063	0.063	0.125	0.03	0.25	0.125	0.063	0.25	0.25		
Transformants															
Trans-HH1609014	128	16	16	16	1	8	8	0.03	0.5	0.25	0.125	0.25	0.5	CTX-M-14	
Trans-XY1608045	256	32	16	16	32	0.5	0.5	0.063	0.25	0.125	0.125	0.125	0.25	CTX-M-14	*aac(6′)-Ib-cr*
Trans-EH1607014	256	16	16	32	32	64	8	0.03	0.125	0.125	0.5	0.125	1	CTX-M-14	*aac(6′)-Ib-cr*
Trans-WH1510002	128	32	16	32	32	64	0.5	0.03	0.063	0.063	0.063	0.063	0.25	CTX-M-9	*aac(6′)-Ib-cr*
Trans-BA1605022	128	32	32	64	1	16	0.5	0.03	0.5	0.063	0.125	0.03	0.5	CTX-M-9, CTX-M-14	*qnrS*, *aac(6′)-Ib-cr*
Trans-QX1604103	128	32	32	1	64	64	16	0.125	0.125	0.125	0.5	0.125	2	TEM-1, CTX-M-14	*aac(6′)-Ib-cr*
Trans-SC1610005	128	16	16	16	1	16	4	0.03	0.5	0.25	0.5	0.5	1	TEM-1, SHV-12, CTX-M-15	*qnrS*, *aac(6′)-Ib-cr*
Trans-XW1609038	128	16	16	16	1	32	16	0.063	0.063	0.063	0.125	0.25	0.5	CTX-M-15	*qnrB*, *aac(6′)-Ib-cr*
Trans-GG1509025	256	32	16	1	0.5	32	1	0.063	0.125	0.125	0.125	0.125	0.25	SHV-12, CTX-M-65	*aac(6′)-Ib-cr*
Trans-GG1610109	256	32	32	32	32	64	16	0.03	0.25	0.063	1	0.25	0.5	CTX-M-15, CTX-M-123, KPC-2, NDM-1	*qepA*, *qnrS*, *aac(6′)-Ib-cr*

### MLST determination

**Fig. 2:**
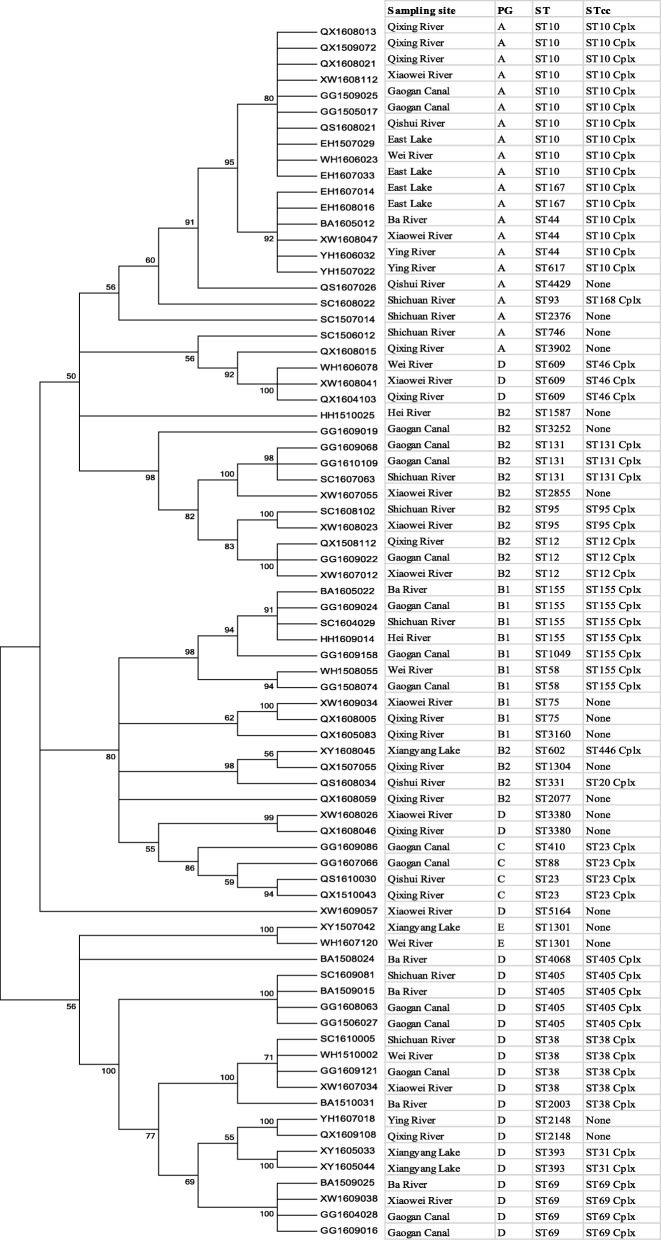
Phylogenetic tree showing the relationship of 76 ESBL-producing *E. coli* isolates. The dendrogram was constructed by using the nucleotide sequences of the seven housekeeping genes (*adk*, *fumC*, *gyrB*, *icd*, *mdh*, *purA* and *recA*) of 76 ESBL-producing *E. coli* isolates from rivers and lakes in Northwest China with the maximum likelihood method. Sampling sites, phylogenetic group (PG), sequence type (ST) and ST clonal complex (STcc) were displayed the right of the dendrogram

The diversity and phylogenetic relationships of the ESBL-producing *E. coli* isolates were evaluated by MLST. MEGA 6.0 software was used to construct the phylogenetic tree for 76 ESBL-producing *E. coli* isolates using the maximum likelihood approach with on the basis of the Tamura-Nei model and seven concatenated housekeeping gene sequences (Fig. 2). The 76 ESBL producers belonged to 37 STs (Fig.1 and Table 1). Among of them, 19 STs were represented by more than two isolates, and the other 18 STs represented a single isolate each. ST10 (*n* = 10) was more prevalent compared with other STs (*P* < 0.001). It is difficult to infer a significant correlation between the water bodies and the STs because of the limited number of ESBL producers. Nevertheless, we found that some ESBL producers from different water bodies shared the same STs, and some STs, e.g., ST10, ST38, ST69, ST405, identified in this study were also found among the *E. coli* isolates from dogs in Shannxi province. Three ST131 isolates were from Shichuan River and Gaogan Canal, which flowed through several cities and villages. Furthermore, the ST131 isolate from Shichuan River simultaneously harbored *bla*_CTX-M-15_ and *bla*_CTX-M-123_; one ST131 isolate from Gaogan Canal harbored *bla*_CTX-M-9_, *bla*_CTX-M-15_, *bla*_CTX-M-123_, *bla*_KPC-2_, *bla*_NDM-1_, *bla*_OXA-2_ as well as PMQR genes *qnrA*, *qnrS* and *aac(6′)-Ib-cr*, while another ST131 isolate from Gaogan Canal harbored *bla*_CTX-M-14_, *bla*_KPC-2_, *bla*_OXA-2_ as well as *qnrB*, *qnrS* and *aac(6′)-Ib-cr*.

## Discussion

The spread of ESBL-producing *E. coli* isolates in the environment, especially in water is worrisome both in developing and developed countries as they pose potential risks to public health [16–18]. Rivers and lakes are usually considered to be of special importance as a reservoir of resistance genes because they can collect the surface waters containing contaminants from different origins, e.g., municipal wastewater, agricultural activities, or the sewage from the hospitals and livestock, which include abundant antibiotic-resistant bacteria. In this study, 2686 *E. coli* isolates were collected from 11 water bodies between March 2015 and November 2016, with 90.9% (10/11) of sampling sites located in Guanzhong region, an economically developed and densely populated area in Shaanxi province. Generally, the prevalence rate of ESBL producers was 2.8%, which was much lower than the prevalence of ESBL producers among the *E. coli* isolated from dogs (24.2%), retail meat (22.3%) and pigs (9.6%, unpublished data from our group) in Shaanxi province [19, 20]. Meanwhile, the frequency of ESBL-producing *E. coli* varied significantly at different sampling sites, and it was more frequently isolated in the Gaogan Canal (6.4%), Qixing River (4.3%) and Xiaowei River (4.2%) compared with Hei River (1.1%) (*P* < 0.01). It is noteworthy that a *bla*_NDM-1_-producing ST131 clone, and four of the five *bla*_OXA-2_-producing isolates were isolated from Gaogan Canal. There is a high probability that the Gaogan Canal, Qixing River and Xiaowei River were contaminated by the wastewater from the hospitals, pharmaceutical manufactures or livestock farms, which are located in or adjacent to cities or rural villages. However, ESBL-producing *E. coli* isolates were seldom detected in the Hei River, Ying Lake and Qishui River, which belong to public water supply source or scenic spots. The results indicate that there is a positive linear relationship between the occurrence of ESBL producers and discharge of wasterwater, such as the sewage of the hospitals and the livestock farms.

It is of particular concern that the majority (84.2%) of 76 ESBL-producing isolates included in this study expressed the MDR phenotype and showed high resistance rates to amoxicillin-clavulanic acid (98.7%), tetracycline (97.3%) and ticarcillin-clavulanic acid (90.8%). Moreover, it is worrisome that most ESBL producers were commonly located on conjugative plasmids that also harbor genes conferring cross-resistance to non-β-lactam antibiotics [21]. Traditionally, phylogroups A and B1 contain commensal isolates, while groups B2 and D are considered to be opportunistic ExPEC isolates. The 76 ESBL-producing *E. coli* isolates surveyed belonged mainly to phylogroups D and A (59.2%), followed by group B2 (19.7%). Normally, virulence factors are ideal targets for determining the pathogenic potential of a given *E. coli* isolate. Most of our ESBL-producing isolates (65.8%) possessed UPEC-related virulence factors, followed by *estA*, which is associated with the ETEC. Our results generally agree with a previous study that found ExPEC as the main pathotype in *E. coli* isolates from other water sources [6]. However, our findings tend to strongly disagree with the previous finding of significantly higher prevalence of ETEC isolates in surface waters of developing countries [22, 23], which may be due to the large differences in the sampling environments. It has been shown that ExPEC isolates can exist as commensals in the guts of healthy animals and humans, where they may gain or lose virulence genes through genetic exchange [6]. Moreover, UPEC isolates, the primary ExPEC associated with urinary tract infections, are also an important source of ESBLs entering the water system [24].

In recent years, CTX-M subtypes of the CTX-M-1 and CTX-M-9 groups have become the most prevalent ESBL-encoding genes among the *E. coli* from clinical and aquatic environments [4]. In the present study, CTX-Ms were represented by seven *bla*_CTX-M_ subtypes that mostly expressed *bla*_CTX-M-14_. Two recent studies in our laboratory revealed that the predominant *bla*_CTX-M_ subtypes in the ESBL-producing *E. coli* isolated from dogs and pigs, respectively, in the Guanzhong region of Shaanxi province [20, 25]. *bla*_CTX-M-15_ and *bla*_CTX-M-14_ were also prevalence in humans in Asia [26]. We identified three isolates that harbored *bla*_CTX-M-65_, which has not been reported before in Northwest China, although it has been frequently reported in other places in China [27–29]. All 76 ESBL-producing isolates were assigned to 37 STs, with ST10 as the most predominant. In contrast to the genetic characteristics of the ESBL-producing *E. coli* isolates from other sources, all the ESBL producers were much more diverse compared to the isolates from pigs and dogs in Shaanxi province. The emergence of clone ST131 represents a major challenge to public health worldwide since it was first discovered in human clinical samples. Subsequently, it has disseminated to various animal species and environments [4]. Our study indicated that three (3.9%, 3/76) ST131 isolates were detected in Shichuan River and Gaogan Canal, of which two ST131 isolates harbored *bla*_CTX-M-15_ and one harbored *bla*_CTX-M-14_, *bla*_KPC-2_ and *bla*_OXA-2_. The previous study suggested that the worldwide pandemic B2-ST131 *E. coli* isolates harboring *bla*_CTX-M-27_-producing have been closely associated with underlying severe infections in human and animal medicine [30]. We also detected four *bla*_CTX-M-27_-producing *E. coli* isolates, although these were not of the ST131 clone. Hence, further studies will need to be performed to explore these isolates, while at the same time, appropriate measures urgently need to be enforced to alleviate the stress posed by antibiotic resistance in the environments.

We found that almost all *bla*_SHV-12_ genes mainly co-existed with non-ESBL gene *bla*_TEM-1_ but not the other β-lactamase genes (Table 1). With respect to PMQR genes, their prevalence among *E. coli* isolates from humans and animals has been described frequently. However, there are few reports on the presence of PMQR genes in the ESBL-producing *E. coli* in water bodies. Our surface water *E. coli* isolates yielded one or more PMQR genes in 71.1% of the ESBL-producing isolates tested, with *aac(6′)-Ib-cr* as the most prevalent (63.2%), which was similar with a previous study in our laboratory that showed *aac(6′)-Ib-cr* as the most prevalent PMQR gene in extended-spectrum cephalosporin-resistant *E. coli* isolates from dogs in Shaanxi [20]. However, a previous study in Heilongjiang province showed that the *oqxAB* gene was the most dominant in the ESBL-producing *E. coli* from piglets [31]. All the PMQR genes co-localized with *bla*_CTX-M_ in our *E. coli* isolates. The emergence of PMQRs indicates that quinolone resistance can also be acquired through horizontal gene transfer, and PMQR genes *qnr* and *aac-(6′)-Ib-cr* were co-transferred with β-lactamase genes, which were confirmed by the conjugation experiments in the present study. Notably in this study, one ST131 isolate from Gaogan Canal simultaneously harbored *bla*_CTX-M-9_, *bla*_CTX-M-15_, *bla*_CTX-M-123_, *bla*_KPC-2_, *bla*_NDM-1_, *bla*_OXA-2_ as well as the PMQR genes *qnrA*, *qnrS* and *aac(6′)-Ib-cr*. To our knowledge, this is the first description of the coexistence of so many resistance genes in one *E. coli* isolate from water. Hence, more studies should be carried out in the future in order to judge if these genes are located on the same plasmid.

## Conclusion

In conclusion, the prevalence of ESBL-producing *E. coli* from the rivers and lakes in Northwest China was 2.8%, and the ExPEC pathotype was the most frequently detected depending on the virulence factor profiles. 76.3% of ESBL producers harbored more than one β-lactamase gene, and *bla*_CTX-M-14_ was the predominant genotype; the most dominant PMQR gene was *aac(6′)-Ib-cr*. The ESBL producers showed a high degree of overlaps in terms of resistance phenotypes, β-lactamases, PMQR genes and other genetic characteristics. The most prevalent sequence type was ST10, and three ST131 clones were detected.

## Abbreviations

EAEC: Enteroaggregative *E. coli*
EIEC: Enteroinvasive *E. coli*
EPEC: Enteropathogenic *E. coli*
ESBL: Extended-spectrum β-lactamase
ETEC: Enterotoxigenic *E. coli*
ExPEC: Extraintestinal pathogenic *E. coli*
PMQR: Plasmid-mediated quinolone resistance
STEC: Shiga toxin-producing *E. coli*
UPEC: Uropathogenic *E. coli*
